# Influence of health interventions on quality of life in seriously ill children at the end of life: a systematic review protocol

**DOI:** 10.1186/s13643-019-1059-8

**Published:** 2019-07-11

**Authors:** Veerle E. Piette, Joachim Cohen, Luc Deliens, Nele Pauwels, Jutte van der Werff ten Bosch, Kim Beernaert

**Affiliations:** 1 End-of-Life Care Research Group, Vrije Universiteit Brussel & Ghent University, Brussels/Ghent, Belgium; 2 Department of Family Medicine and Chronic Care, Vrije Universiteit Brussel, Laarbeeklaan 103, 1090 Brussels, Belgium; 3 Department of Public Health and Primary Care, Ghent University, C. Heymanslaan 10, 9000 Ghent, Belgium; 4 Department of Paediatrics, University Hospital Brussels, Vrije Universiteit Brussel, Laarbeeklaan 101, 1090 Brussels, Belgium; 5 Knowledge Centre for Health Ghent, Ghent University, C. Heymanslaan 10, 9000 Ghent, Belgium

**Keywords:** Children, Pediatrics, End of life, Seriously ill, Quality of life, Health intervention, Review, Systematic, Symptom control

## Abstract

**Background:**

Seriously ill children suffer from numerous symptoms at the end of their lives, including pain, anxiety, and restricted communication. There are currently no comprehensive overviews of which health interventions have proven benefits and which have proven detrimental effects on the quality of life of children in an end-of-life context. In order to identify potential quality indicators to eventually improve care, a systematic review of available evidence is needed. The aim of the current systematic review will be to make an overview of the influence of health interventions on associated outcomes related to quality of life at the end of life in seriously ill children.

**Methods:**

A systematic search will be conducted in MEDLINE, Embase, CENTRAL, CINAHL, and Web of Science. We will include quantitative empirical designs looking into the influence of a health intervention on (proxies of) quality of life at the end of life in seriously ill children. Three independent authors will review titles and abstracts and screen full texts against eligibility criteria. One reviewer will carry out full data extraction and quality assessment, and a 20% random sample will be extracted and assessed by two independent reviewers. We will use the QualSyst Tool for assessment of the quality of the included studies (QualSyst Tool) for quality assessment; overall strength of the body of evidence will be assessed using the Grading of Recommendations Assessment, Development, and Evaluation (GRADE) approach. An overview table of health interventions will be discussed through narrative synthesis. Should sufficient homogeneous publications arise, we will perform meta-analyses with a random-effects model. Our protocol adheres to the Preferred Reporting Items for Systematic Review and Meta-Analysis Protocols (PRISMA-P) checklist for study protocols.

**Discussion:**

As part of a larger project, we will use the results of this review to identify a first set of quality indicators for the care for children at the end of life. Reviewing the current span of evidence and identifying research gaps will uncover future research priorities into the care for children at the end of life.

**Systematic review registration:**

PROSPERO CRD42018105109

**Electronic supplementary material:**

The online version of this article (10.1186/s13643-019-1059-8) contains supplementary material, which is available to authorized users.

## Background

Despite medical advancements and intensive research into therapy and treatment, some children’s illnesses such as cancer, neurological conditions, and other progressive, life-threatening disorders remain incurable even in developed countries. Worldwide, children with progressive, serious illnesses estimate one-third of yearly child mortalities [1]. International mortality rates for these disorders vary between hundreds of children per year for small-population countries and thousands of children per year for largely populated countries [1]. Moreover, prevalence of children dying due to serious illness seems to be on the rise: an increase of 25 to 32 per 10,000 population was reported in England between 2000 and 2010 [2].

The symptom burden at the end of life in these children is found to be very high [3–6]. Symptoms and care needs at the end of life have been studied extensively for malignancies [3, 5, 6], with pain, fatigue, dyspnea, and anorexia often cited, but less so for other disease trajectories [4], where the scarce research seems to show distinct symptoms at the end of life such as numbness and breathing problems [4]. The most frequently reported symptoms are not only physical, such as pain and dyspnea, but also psychological, such as nervousness and worrying [4–6]. Due to high symptom burden, many researchers and clinicians advocate more extensive development of supportive and end-of-life care for this population.

According to the American Academy of Pediatrics, family-centered, child- and disease-specific supportive care for children should be broadly available, and well-integrated within the disease trajectory [7]. However, there are indications children often do not receive supportive care when dying, among which care at the end of life. A recent systematic review indicated almost half of children with cancer did not receive palliative care at the end of life [8], even though palliative care is indicated to improve patient outcomes [9, 10]. Children tend to receive intense aggressive care in the last weeks of life [11–14], while symptom control for pain, fatigue, and dyspnea has been shown to be insufficient [5]. Increased attention for supportive care measures for children in recent decades may have spurred improvement: a 1997–2004 US follow-up cohort of children suffered less from pain and dyspnea than a cohort from 1990 to 1997, possibly due to improvement in end-of-life care management for children [5]. Nevertheless, various studies have reported ongoing issues for children at the end of life such as low accessibility to pediatric palliative care services [15], lack of communication between care professionals [16], insufficient resources and training [17], and absence of children-specific quality measures [11, 18]. Children globally lack access to supportive care measures [19], and while low access to palliative care is associated in general with low-income countries [20, 21], also high-income countries such as Canada can struggle with accessibility to supportive care due to, e.g., wide geography [22]. Challenges for supportive care for children at the end of life differ overall between high-, middle-, and low-income countries, with low-income country challenges often relating to the lack of resources and finances, and high-income countries focusing on improvement and continuity of care [22].

To further improve quality of life at the end of life for seriously ill children, we need more insight into the health interventions that influence quality of life at the end of life. However, there is a lack of knowledge about what health interventions, e.g., medication or palliative care, can influence quality of life at the end of life exactly, e.g., outcomes such as pain or anxiety. Knowing what health interventions are appropriate and inappropriate will enable the development of quality indicators of this care. Quality indicators are measurable items referring to the outcomes, processes, or structure of care. They can be used to monitor, assess, benchmark, and eventually improve appropriate and inappropriate end-of-life care for children. Multiple quality indicators were already established for adult end-of-life care, and one such indicator for inappropriate care for cancer patients is a blood transfusion in the last month before death [23].

A first step in developing validated sets of quality indicators is to systematically review the literature describing the health interventions that influence quality of life in seriously ill children at the end of life. To our knowledge, there is no systematic overview to date of known health interventions that have an influence—whether negative, neutral, or positive—on quality of life at the end of life in children’s care. Available reviews in seriously ill children are limited in that they mainly focus on health interventions and associated quality-of-life outcomes in a curative phase, but not terminal or end-of-life phase [3, 24, 25], or they focus only on the association between one health intervention and quality of life, such as the identification of benefits of specialized pediatric palliative care services [4] and do not provide an overview of other health interventions at the end of life. Individual studies of various health interventions and associations or impact on quality of life at the child’s end of life are available but have not yet been systematically reviewed, summarized, or assessed for quality or bias. This makes it difficult to scan the entire body of evidence for quality, gaps, and effects for subgroups. For instance, the influence of health interventions could differ in terms of disease and age—differences are likely to arise between cancer patients and those suffering of neurological disorders, or between toddlers and teenagers. Therefore, we will conduct this comprehensive systematic review on the influence of various health interventions for seriously ill children at the end of life, identifying all available evidence that associates health interventions with quality of life at the end of life in seriously ill children in quantitative empirical designs. The results of the systematic review will become one pool of candidate quality indicators from which final quality indicators will be chosen and face-validated using the RAND/UCLA (Research ANd Development corporation/University of California Los Angeles) Appropriateness Method, a modified Delphi panel method.

This study protocol has drawn upon the Preferred Reporting Items for Systematic Review and Meta-Analysis Protocols (PRISMA-P) guidelines for the reporting of systematic review protocols. Any updates will be provided through the PROSPERO registry of systematic reviews.

## Methods

### Concepts and definitions

For the purpose of the present study, the following definitions will be used: (1) quality of life is defined as all measures or proxies of quality of life, care, or dying at the end of life, containing six domains (physical health, psychological health, independence level, social relations, environment, spirituality/religion/personal beliefs [26]); (2) a health intervention is any “act performed for, with or on behalf of a person or population whose purpose is to assess, improve, maintain, promote or modify health, functioning or health conditions” [27] which also includes decisions about treatment, place of care, place of death, etc.; (3) seriously ill children are defined as people, who are suffering from a progressive life-threatening disorder, excluding acutely ill children at the end of life, i.e., those whose illness is due to trauma, suicide, or other unforeseen complications not related to a syndrome or illness; and (4) end of life is the period preceding the death of the child during the last months, weeks, or days. To maximize the sensitivity of our search, we will not delineate a cut-off point in months or days for the end-of-life period, nor will we define a finite group of disorders for the same reason.

### Eligibility criteria

#### Study design

##### Inclusion

We will include all quantitative empirical studies that measure the influence of a health intervention on quality of life or proxies for quality of life affecting more than 1 child.

We will include interventional, observational, and survey designs. We will include the following types of interventional designs: pre-post study designs, non-randomized trials, and randomized controlled trials. Observational designs cannot show a causal relationship between an intervention and quality-of-life outcome, yet by showing associations can provide indications for the evaluation of interventions in a field where evidence is scarce. Therefore, the following types of observational designs will be included: cohorts, case-control designs, and cross-sectional designs. Lastly, we will include survey designs with quantifiable results (e.g., measured on a Likert scale). Both prospective and retrospective designs will be included.

##### Exclusion

We will exclude protocol papers, meta-analyses, literature reviews, studies with a single case design, qualitative research, and gray literature. Meta-analyses and reviews will be excluded because the individual studies should already appear in our selection. Qualitative and gray research will be excluded because the review of qualitative and gray literature requires different selection and evaluation criteria that we believe are better discussed in a separate review.

#### Population

##### Inclusion

We will include all publications that aim to study children at the end of life. The publication should include more than one seriously ill child equal to or between 1 and 17 years, a mean or median age equal to or between 1 and 17 years for a group of seriously ill children when no individual ages are provided, or address seriously ill children in general by means of terms such as “children,” “adolescents,” “Pediatric Intensive Care Unit,” or other terms relating to children, and describe them with terms such as “terminal,” “seriously ill,” “near death,” “dying,” or other medical terms indicating the end of life. Children’s age will not have to be mentioned in the title or abstract for a study to be eligible for selection. When it is unclear if the population is at the end of life, this will be discussed between reviewers, and if necessary, a pediatrician involved in the project will decide whether the population is at the end of life.

#### Intervention or exposure

##### Inclusion

We will include all publications that assess at least one health intervention—e.g., medication, treatments, programs (trials, Advanced Care Planning; palliative and psychosocial care; decisions on source of care, place of care, place of death)— provided to the child at the end of life, indicated explicitly by terms such as “end of life,” “dying” or similar phrases, or medical terms indicating end of life.

#### Outcome

##### Inclusion

We will include all publications with at least one proxy of quality of life akin to symptom assessment, relief, or intensity; treatment burden, intensity, or toxicity; disability and psychosocial status; adverse symptoms; and perceptions on quality of life of the child.

##### Exclusion

We will exclude all outcomes akin to demographics, health resource use, conformity to guidelines, quality of life of the parent, medical staff, or people involved other than the child. These measures will be excluded as the measures are conceptually too remote from the concept of quality of life, even if the publication itself indicates the measure as a proxy. These measures will not be excluded when they are present in the form of interventions, but only when they appear in the form of quality-of-life outcomes, i.e., as a proxy of quality of life of the child.

Outcomes that are not mentioned here and initiate a discussion between the reviewers will be discussed between the reviewers, and if necessary, with a third reviewer.

#### Other

##### Inclusion

We will include all publications

1. Published from 1/1/2000 onwards. Papers before 2000 will not be included as practices before 2000 are likely to differ considerably from contemporary practices, due to the ongoing developments in children’s care, palliative care, and general medicine. We based our choice of cut-off on the emergence of the first research on pediatric palliative care [28]. Pediatric palliative and end-of-life care is a recently emerging field and attention was only brought to the pressing issues children at the end of life face from 2000 onwards. Not only is there hardly any literature available on the topic before 2000, but we also believe an increased awareness with regard to pediatric palliative care signals a different medical climate and should therefore not be part of the evidence base we are constructing to underlie quality indicators for our current medical care system that is much more sensitive to supportive care measures.
2. With the title and abstract published in English. Full texts in other languages than English, Dutch, French, and German will be translated by a native speaker when needed. No language restrictions were imposed.

### Systematic search

#### Search strategy

The search strategy will be structured around four distinct blocks: population (children), care at the end of life, quality of life at the end of life, and design of the study. The PubMed database and syntax will be used as a starting point for construction and validation. For construction, the search blocks will be based on previous search filters, some validated, as well as Medical Subject Headings (MesH) terms and free text words. Experts on care for children at the end of life (i.e., the authors of the review) as well as relevant papers resulting from a scoping review will be consulted for MesH terms and relevant free text words to augment and/or refine the strategy. The iterative process of construction will be guided and checked by an information specialist. The help of an information specialist, who in our case is specialized in the development of search strategies, database searches, and systematic review methodology among other things, is crucial in constructing and revising a search strategy. Information specialists or professionals have special knowledge in quality health information resources. They have a direct impact on the quality of patient care, helping physicians, allied health professionals, administrators, students, faculty, and researchers stay abreast of and learn about new developments in their fields. Using materials and tools that range from traditional print journals to electronic databases and the latest mobile devices, information specialists use innovative strategies to access and deliver important information for patient care, research, and publication. The information professional might have a library background/education or might have a health profession or researcher background. An information specialist will provide guidance and support in all steps of a systematic review including the construction of a very sensitive search strategy for this systematic review.

For validation of the search strategy, a validation set of records will be constructed by hand-searching six volumes of pediatric medical journals between 2000 and 2018 that adhere to our eligibility criteria by two reviewers to obtain a gold standard. The six volumes will be picked randomly using Excel.

The PubMed search strategy will be translated to the other databases in collaboration with an information specialist. Independent information specialists will peer-review the strategies by email correspondence.

A preliminary version of the validated search strategy, reference set, and validation set for MEDLINE using PubMed are available in the additional documents (see Additional files 1 and 2). The search strategy adheres to the Peer Review of Electronic Search Strategies (PRESS) criteria for electronic search strategies as stated in the 2015 Guideline Statement [29].

#### Sources

The systematic search will be conducted in MEDLINE (using the PubMed interface), Embase (using the embase.com interface), CENTRAL, CINAHL (using the EBSCO interface), and Web of Science.

#### Publication selection

Papers will be selected by three independent researchers, following Cochrane guidelines to reduce bias with a two-step screening process. When a publication is identified as potentially relevant, based on its title and/or abstract, by the eligibility criteria described above, a full-text review will be done by the same three independent authors applying the same eligibility criteria. If discrepancies should arise between assessors, they will be resolved by consensus. An additional author will be consulted in cases where no agreement between the two assessors can be reached. The online software program Covidence will be used to keep track of the selection process, which was developed by the Cochrane foundation explicitly for the use of systematic reviews, to upload all references of selected articles via Endnote and extract all titles, abstracts, and pdfs. It allows to easily keep track of exclusion and inclusion by all reviewers.

For the full-text review, we will look for the selected articles online, utilise our university library services to obtain articles requiring subscription, and/or contact colleagues and authors to obtain the full text. When no full text of the article can be obtained this way, the record will be excluded. In accordance with the PRISMA guidelines [30], the rationale for rejection of each paper will be recorded during the full-text review phase. The reference lists of all identified publications will then be screened for additional relevant publications. The corresponding author of each publication and known experts in the field of care for children at the end of life will be contacted for possible additional publications eligible for inclusion. Assessment for the inclusion of included papers in a possible meta-analysis will be done independently by the same three reviewers.

### Data extraction and quality assessment

#### Data extraction

A pilot-tested data extraction form will be used to systematically extract data. The extraction form will be tested with two studies by two reviewers prior to the full extraction process. Discrepancies will be resolved through discussion between two reviewers and, when necessary, by consulting an additional co-author reviewer. The following data will be extracted by one researcher, with a 20% sample reviewed by another researcher: title; authors; date of publication (month, day); year of publication; journal; country (where data collection took place); aim/research question(s) (as stated in the study); start and end of intervention or exposure (in days before death); duration of intervention (in days); setting (where health intervention took place); population; participants (who + number); children’s age (mean, median, range, and/or interquartile range (IQR)); children’s age group (0–1, 1–5, 6–9, 10–14, 15–17, 18+); children’s illness (as stated in the study); children’s illness category (cancer, neurological disorder, or other); language of article; definition of end of life; indications of end of life throughout the article; reported health intervention(s) and quality of life; measurement of the health intervention; measurement of quality of life (proxy); definer of quality of life (proxy); study design; quality score based on the QualSyst tool (as described below); reported influence of health intervention; availability of separate results for target age group; and availability of separate results for children at the end-of-life stage.

#### Risk of bias and quality assessment

The quality of each individual study will be assessed with the QualSyst checklist for assessing the quality of quantitative studies. The 14-item checklist will be used to assess research questions and objectives, study design, subject and comparison group selection and characteristics, interventional allocation, definitions of outcomes, sample size, analytic methods, confounding, and reports of results. We will calculate interrater agreement between the reviewers for a 20% sample. For assessment of the full body of evidence, i.e., for each intervention and outcome, we will utilize the Grading of Recommendations Assessment (GRADE) approach [31]. GRADE is a step-wise method to assess the certainty of evidence in the synthesis of scientific literature, such as reviews and guidelines, and is commonly used for the appraisal of effects found of health interventions in health care and the public health domain. The method is used to rate evidence per outcome and starts with a high rating for RCTs and low rating for all other study designs. Eight criteria, such as risk of bias or large magnitude of effect, are then used to either upgrade or downgrade the final rating to high, moderate, low, or very low quality of evidence. This way, the method can indicate how certain we can be of an effect and whether reliable recommendations for practice in health care can be made based on the evidence that is available.

The QualSyst tool will be used to obtain a quality assessment of each study, and the GRADE approach will then be used to grade the quality of the body of evidence for each health intervention.

One reviewer will carry out full data extraction and quality assessment (QualSyst and GRADE), and a 20% random sample will be extracted and assessed by two independent reviewers. Discrepancies will be resolved through consensus with a third reviewer.

#### Data synthesis

We will create an overview table of all health interventions and associated quality of life-related outcomes at the end of life in children. We will include health intervention, health intervention category (e.g. medication, treatment), quality-of-life outcome, quality-of-life category (physical health, psychological health, independence level, social relations, environment, spirituality/religion/personal beliefs [26]); the number of studies (by health intervention and quality-of-life outcome); definer(s), age group; disease group; timing before death in days; reported influence (descriptive); whether or not the influence on the quality of life of other persons involved was measured as well; GRADE score; and additional comments.

#### Statistical analysis

A meta-analysis will be performed if sufficient homogeneous studies of similar outcome, design and measure are found. As we expect to find a variety of health interventions as well as a variety of quality of life-related outcomes, two independent reviewers will first discuss clinical and methodological heterogeneity for all similar health interventions. The two reviewers will discuss outcomes and their methodology for all similar health interventions with two or more outcomes. Clinical and methodological heterogeneity will be assumed when a different quality-of-life outcome is assessed for the same intervention in terms of symptomology (e.g., pain versus anxiety) or when a different measurement is used for the outcome (e.g., pain scale versus nurse evaluation of pain). If clinical and methodological homogeneity is present, we will assess statistical heterogeneity. Statistical heterogeneity will be assessed with the chi-squared (*χ*2) test and inconsistency index (*I*^2^). In agreement with the Cochrane Collaboration threshold recommendations for the assessment of heterogeneity, we will not perform meta-analysis when *p* value is below 0.1 or the *I*^2^ statistic is higher than 75% [32]. If no statistical heterogeneity seems to be present, we will perform a random effects model with a 95% confidence interval. If more than ten studies measure similar outcomes and interventions, sensitivity analyses will be conducted to examine the robustness of results resulting from meta-analysis. To do so, we will conduct separate meta-analyses without the studies with high risk of bias (selection based on GRADE) and will compare these analyses with the meta-analysis with all studies included regardless of risk of bias. In case more than ten studies assessing the same quality-of-life outcome for a similar health intervention are available, publication bias will be evaluated using a funnel plot, assessing plot symmetry of the available effects. If sufficient age and disease characteristics are given within the studies used for meta-analysis, numerical subgroup analyses will be done for these factors. Data for subgroup analyses will be obtained from demographic data included in the study.

We will use the Cochrane meta-analysis software Review Manager 5 (Revman 5) for all meta-analysis calculations. Outcomes are expected to be continuous as well as dichotomous. We will report continuous outcomes as means, the difference in means, and their 95% confidence intervals. When mean is not given, other summary statistics available such as median will be reported. We will report dichotomous outcomes as odds ratios or percentages, and their 95% confidence intervals.

In case no meta-analysis is possible, a narrative synthesis will be done, in which we will include a discussion of relevant factrs such as age, disease group, and definers of quality of life.

## Discussion

To our knowledge, no systematic review of studies has yet been conducted that looks into the influence of health interventions on quality of life at the end of life in seriously ill children. This systematic review will add to the construction of a reliable and valid evidence base to be utilized in children’s end-of-life research and in health care policy to improve quality of life and care. As part of a larger project on quality indicators, the review is a primary step in the construction of a set of quality indicators for care for children at the end of life for multiple disease groups, pending face-validation, and expert consensus. Quality indicators are regarded as a valid tool to monitor care standards with the use of population-level data [33]. We will apply the quality indicators to population-level data for deceased seriously ill children in Flanders and compare results on quality indicators for the different populations, settings, and regions. Additionally, we will benchmark care between different health regions for all indicators to set performance standards and norms that can eventually lead to actual improvement of care. This review protocol is published to allow other researchers to compare previously established methods to the final review, promoting quality adherence, and to facilitate future updates of the review to keep quality indicators up-to-date by identifying any new potential quality indicators arising in future quantitative empirical studies.

Expected limitations of the review are differences in patient and disease characteristics, and the exclusion of qualitative studies and process- and structure-related outcomes. Care for children at the end of life is a heterogeneous field in terms of disease etiology, progression, and age—for instance, the developmental gap between young children and adolescents is likely to lead to differences in the influence of health interventions. Due to limited patient availability, study samples are often likely to contain children with various etiologies and in various disease stages. We will ensure subgroup analyses in our narrative and numerical analyses whenever possible. Our exclusion criteria omit qualitative studies and process- and structure-related quality-of-life outcomes, and this omission may bias the evaluation of the evidence base. However, we believe the analysis of the qualitative studies requires a distinctly different approach than the analysis of the quantitative studies, and systematic reviews are regularly limited to the synthesis of quantitative papers. As quantitative outcomes will already provide sufficient material for synthesis, qualitative studies are therefore better discussed and synthesized in a separate study. We acknowledge, however, that the addition of qualitative designs can only broaden our understanding of the current evidence base of the influence of health interventions on quality of life in seriously ill children, and therefore, we plan on conducting an additional scoping review that summarizes outcomes found in qualitative studies. We urge that process and structure outcomes be looked into systematically as well, as these outcomes could also significantly implicate end-of-life care in children at the end of life.

## Additional files

Additional file 1: Reference and validation set for construction of the MEDLINE search strategy.

Additional file 2: Search strategy for MEDLINE (PubMed interface).

## Data Availability

Not applicable

## Abbreviations

ACP: Advanced Care Planning
CI: Confidence interval
CINAHL: Cumulative Index to Nursing and Allied Health Literature
GRADE: Grading of Recommendations Assessment, Development, and Evaluation
MeSH: Medical Subject Headings
PICU: Pediatric Intensive Care Unit
PRESS: Peer Review of Electronic Search Strategies
PRISMA: Preferred Reporting Items for Systematic Review and Meta-Analysis Protocols
PROSPERO: International Prospective Register of Systematic Reviews
QOL: Quality of life
