# Supplementary material for: Influence of health interventions on quality of life in seriously ill children at the end of life: a systematic review protocol
Source: Syst Rev. 2019 Jul 11;8:165. doi: 10.1186/s13643-019-1059-8 (PMC6621986; doi:10.1186/s13643-019-1059-8)
Supplement: Supplementary file 1 — Reference and validation set for construction of the MEDLINE search strategy. [file 13643_2019_1059_MOESM1_ESM.pdf]

Reference set for developing search string in MEDLINE (PubMed interface)

[1] Anghelescu DL, Snaman JM, Trujillo L, Sykes AD, Yuan Y, Baker JN. Patient-controlled analgesia at the end of life at a pediatric oncology institution. Pediatric blood and cancer. 2015;62:1237-1244.
[2] Bolt EE, Flens EQ, Pasman HRW, Willems D, Onwuteaka-Philipsen BD. Physician-assisted dying for children is conceivable for most Dutch paediatricians, irrespective of the patient's age or competence to decide. Acta Paediatrica. 2017;106:668-675.
[3] Burns J, Jackson K, Sheehy KA, Finkel JC, Quezado ZM. The use of dexmedetomidine in pediatric palliative care: a preliminary study. Journal of palliative medicine. 2017;20:779-783.
[4] Flerlage JE, Baker JN. Methylnaltrexone for opioid-induced constipation in children and adolescents and young adults with progressive incurable cancer at the end of life. Journal of palliative medicine. 2015;18:631-633.
[5] Van der Geest IM, Heuvel-Eibrink MM, Zwaan CM, Pieters R, Passchier J, Darlington ASE. Participation in a clinical trial for a child with cancer is burdensome for a minority of children. Acta Paediatrica. 2016;105:1100-1104.
[6] Korzeniewska-Eksterowicz A, PrzysloL, Fendler W, Stolarska M, M lynarski W. Palliative sedation at home for terminally ill children with cancer. Journal of pain and symptom management. 2014;48:968-974.
[7] Mak KS, Lee SW, Balboni TA, Marcus KJ. Clinical outcomes and toxicity following palliative radiotherapy for childhood cancers. Pediatric blood and cancer. 2018;65.
[8] McCulloch R, Sattar M, Henderson EM, Lane ME, Bluebond-Langner M. Use of buccal morphine in the management of pain in children with life-limiting conditions: Results of a laboratory study. Palliative medicine. 2018;32:554-558.
[9] Osenga K, Postier A, Dreyfus J, Foster L, Teeple W, Friedrichsdorf SJ. A comparison of circumstances at the end of life in a hospital setting for children with palliative care involvement versus those without. Journal of pain and symptom management. 2016;52:673-680.

Selection based on initial scoping review, recommended articles, and expert opinion (information specialist and content experts).

Validation set for developing search string in MEDLINE (PubMed interface)

[1] Friedrichsdorf SJ, Postier A, Dreyfus J, Osenga K, Sencer S, Wolfe J. Improved quality of life at end of life related to home-based palliative care in children with cancer. Journal of palliative medicine. 2015;18:143-150.
[2] Flerlage JE, Baker JN. Methylnaltrexone for opioid-induced constipation in children and adolescents and young adults with progressive incurable cancer at the end of life. Journal of palliative medicine. 2015;18:631-633.
[3] Korzeniewska-Eksterowicz A, Przys lo L, Fendler W, Stolarska M, M lynarski W. Palliative sedation at home for terminally ill children with cancer. Journal of pain and symptom management. 2014;48:968-974.

Selection of validation set based on eligibility criteria while searching the following volumes: Pediatrics Volume 139 and 140 (2017), Palliative medicine Volume 29 (2015), Journal of Palliative Medicine Volume 18 (2015), Pain and symptom management Volume 47 (2014), Pediatric Blood and Cancer Volume 54 and 55 (2010), Acta paediatrica Volume 94 (2005).
