# Supplementary material for: Influence of health interventions on quality of life in seriously ill children at the end of life: a systematic review protocol
Source: Syst Rev. 2019 Jul 11;8:165. doi: 10.1186/s13643-019-1059-8 (PMC6621986; doi:10.1186/s13643-019-1059-8)
Supplement: Supplementary file 2 — Search strategy for MEDLINE (PubMed interface). [file 13643_2019_1059_MOESM2_ESM.pdf]

Validated search string for MEDLINE (PubMed interface)

1. Pediatrics
child [mh] OR pediatrics [mh:noexp] OR adolescent [mh] OR minors [mh] OR toddler [tiab] OR toddlers [tiab] OR minors [tiab] OR boy [tiab] OR boys [tiab] OR girl [tiab] OR girls [tiab] OR kid [tiab] OR kids [tiab] OR child [tiab] OR child's [tiab] OR children [tiab] OR children's [tiab] OR childhood [tiab] OR schoolchild [tiab] OR schoolchildren [tiab] OR school age [tiab] OR school aged [tiab] OR school ager [tiab] OR school agers [tiab] OR school ages [tiab] OR adolescence [tiab] OR adolescent [tiab] OR adolescent's [tiab] OR adolescents [tiab] OR adolescents' [tiab] OR underage* [tiab] OR pediatric [tiab] OR pediatrician [tiab] OR pediatricians [tiab] OR pediatrics [tiab] OR paediatric [tiab] OR paediatrician [tiab] OR paediatricians [tiab] OR paediatrics [tiab]
2. End of life
Terminal Care [mh] OR palliative care [mh] OR terminally ill [mh] OR hospice care [mh] OR palliative medicine [mh] OR Hospices [mh] OR right to die [mh] OR respite care [mh] OR hospice and palliative care nursing [mh] OR euthanasia [mh] OR suicide, assisted [mh] OR terminal care [tiab] OR terminal disease [tiab] OR terminal diseases [tiab] OR terminal disorder [tiab] OR terminal disorders [tiab] OR terminal cancer [tiab] OR terminal cancers [tiab] OR terminal nature [tiab] OR terminal ilness [tiab] OR terminal ilnesses [tiab] OR terminal remission [tiab] OR terminal remissions [tiab] OR terminal phase [tiab] OR terminal phases [tiab] OR terminally ill [tiab] OR palliative [tiab] OR palliatively [tiab] OR end of life [tiab] OR end-of-life [tiab] OR eolc [tiab] OR EOL [tiab] OR comfort care [tiab] OR assisted suicide [tiab] OR physician-assisted dying [tiab] OR euthanasia [tiab] OR last month of life [tiab] OR last months of life [tiab] OR last days of life [tiab] OR last day of life [tiab] OR life limiting condition [tiab] OR life limiting conditions [tiab]
3. Proxies for quality of life
Patient Acceptance of Health Care [mh] OR Quality-Adjusted Life Years [mh] OR Symptom Assessment [mh] OR Behavioral Symptoms [mh] OR anxiety [mh] OR dyspnea [mh] OR diarrhea [mh:noexp] OR fatigue [mh] OR constipation [mh] OR vomiting [mh] OR "Outcome Assessment (health care)" [mh] OR nausea [mh] OR depression [mh] OR Quality of life [mh] OR pain [mh] OR appetite [tiab] OR appetite [mh] OR Health status [mh:noexp] OR "health status disparities" [mh] OR Health status indicators [mh] OR Quality adjusted life years [mh] OR Treatment outcome [mh] OR Quality Improvement [mh] OR Patient Satisfaction [mh] OR "Standard of Care" [mh] OR Quality of life [tiab] OR QOL [tiab] OR HRQL [tiab] OR HRQOL [tiab] OR Quality adjusted life year [tiab] OR Quality adjusted life years [tiab] OR HRQL [tiab] OR QALY [tiab] OR QALYs [tiab] OR Life quality [tiab] OR Wellbeing [tiab] OR Well-being [tiab] OR pain [tiab] OR nausea [tiab] OR vomit [tiab] OR vomiting [tiab] OR constipation [tiab] OR diarrhea [tiab] OR dyspnea [tiab] OR fatigue [tiab] OR anxiety [tiab] OR depression [tiab]
4. Design
Clinical Study [ptyp] OR Clinical Trial [ptyp] OR Clinical Trial, Phase I [ptyp] OR Clinical Trial, Phase II [ptyp] OR Clinical Trial, Phase III [ptyp] OR Clinical Trial, Phase IV [ptyp] OR comparative Study [ptyp] OR Controlled Clinical Trial [ptyp] OR Evaluation Studies [ptyp]

OR Multicenter Study [ptyp] OR Observational Study [ptyp] OR Pragmatic Clinical Trial [ptyp] OR Randomized Controlled Trial [ptyp] OR Technical Report [ptyp] OR Twin Study [ptyp] OR "non-randomized controlled trials as topic" [mh] OR Health surveys [mh:noexp] OR "adaptive clinical trials as topic" [mh] OR "pragmatic clinical trials as topic" [mh] OR "compassionate use trials" [mh] OR "random allocation" [mh] OR "double-blind method" [mh] OR "single-blind method" [mh] OR "comparative effectiveness research" [mh] OR control groups [mesh] OR "Randomized Controlled Trials" [mh] OR Surveys and Questionnaires [mh] OR "historically controlled study" [mh] OR "controlled before-after studies" [mh] OR "follow-up studies" [mh] OR "sampling studies" [mh] OR "longitudinal studies" [mh] OR "preliminary data" [mh] OR "interrupted time series analysis" [mh] OR "empirical research" [mh:noexp] OR "nursing administration research" [mh] OR Early Termination of Clinical Trials [mh] OR "psychopharmacology" [mh] OR "population surveillance" [mh] OR "multicenter studies as topic" [mh] OR "drug evaluation" [mh] OR "outcome assessment (healthcare)" [mh] OR "observational studies as topic" [mh] OR "clinical studies as topic" [mh] OR "national longitudinal study of adolescent health" [mh] OR "case-control studies" [mh] OR "cohort studies" [mh] OR "cross-over studies" [mh] OR "retrospective studies" [mh] OR "feasibility studies" [mh] OR "pilot projects" [mh] OR clinical study [tiab] OR clinical studies [tiab] OR Comparative study [tiab] OR comparative studies [tiab] OR Evaluation Studies[tiab] OR Evaluation study [tiab] OR Multicenter Study[tiab] OR Multicenter Studies [tiab] OR Observational Study [tiab] OR Observational Studies [tiab] OR Twin Study [tiab] OR Twin Studies [tiab] OR trial [tiab] OR trials [tiab] OR random [tiab] OR randomized [tiab] OR randomized [tiab] OR controlled [tiab] OR controled [tiab] OR multicenter [tiab] OR longitudinal [tiab] OR case-control [tiab] OR case-controls [tiab] OR cohort [tiab] OR cohorts [tiab] OR single-blind [tiab] OR double-blind [tiab] OR "cross-sectional studies"[mh] OR cross-sectional [tiab] OR retrospective [tiab] OR follow-up [tiab] OR non-randomized [tiab] OR pilot study [tiab] OR survey [tiab] OR questionnaire [tiab] OR surveys [tiab] OR questionnaires [tiab] OR case study [tiab] OR laboratory study[tiab]

With time limit: 1/1/2000 - 11/07/2018

Based on (validated) existing search strings with irrelevant terms and truncators removed and literature [1, 2, 3, 4, 5, 6, 7], reference set, and expert opinion (information specialist and content experts).

References

[1] E Leclercq, MM Leeang, E van Dalen, LC Kremer. Validation of search filters for identifying pediatric studies in PubMed. The Journal of pediatrics. 2013;162:629-634.
[2] RM Sladek, J Tieman, DC Currow. Improving search _lter development: a study of palliative care literature. BMC Medical Informatics and Decision Making. 2007;7:18.
[3] CL Von Baeyer, LJ Spagrud. Systematic review of observational (behavioral) measures of pain for children and adolescents aged 3 to 18 years. Pain. 2007;127:140-150.

[4] P Muris, H Merckelbach, B Mayer, A van Brakel, S Thissen, V Moulaert, B Gadet. The screen for child anxiety related emotional disorders (SCARED) and traditional childhood anxiety measures. Journal of Behavior Therapy and Experimental Psychiatry. 1998;29:327-339.
[5] S Paisley, A Booth, S Mensinkai. Chapter 12: health-related quality of life studies. In: Etext on Health Technology Assessment (HTA) Information Resources. 2005.
[6] J Wolfe, HE Grier, N Klar, SB Levin, JM Ellenbogen, S Salem-Schatz, EJ Emanuel, JC Weeks. Symptoms and sffering at the end of life in children with cancer. New England Journal of Medicine. 2000;342:326-333.
[7] KA Robinson, K Dickersin. Development of a highly sensitive search strategy for the retrieval of reports of controlled trials using PubMed. International journal of epidemiology. 2002;31:150-153.
